# Enhancing Attention by Synchronizing Respiration and Fingertip Pressure: A Pilot Study Using Functional Near-Infrared Spectroscopy

**DOI:** 10.3389/fnins.2019.01209

**Published:** 2019-11-12

**Authors:** Yi-Lei Zheng, Dang-Xiao Wang, Yu-Ru Zhang, Yi-Yuan Tang

**Affiliations:** ^1^State Key Laboratory of Virtual Reality Technology and Systems, Beihang University, Beijing, China; ^2^Peng Cheng Laboratory, Shenzhen, China; ^3^Beijing Advanced Innovation Center for Biomedical Engineering, Beihang University, Beijing, China; ^4^Department of Psychological Sciences, Texas Tech University, Lubbock, TX, United States

**Keywords:** sustained attention, cognitive improvement, meditation, functional near-infrared spectroscopy, respiration, fingertip pressure

## Abstract

Sustained attention is a fundamental ability ensuring effective cognitive processing and can be enhanced by meditation practice. However, keeping a focused meditative state is challenging for novices because involuntary mind-wandering frequently occurs during their practice. Inspired by the potential of force-control tasks in invoking internal somatic attention, we proposed a haptics-assisted meditation (HAM) to help reduce mind-wandering and enhance attention. During HAM, participants were instructed to maintain awareness on the respiration and meanwhile adjust bimanual fingertip pressures to keep synchronized with the respiration. This paradigm required somatosensory attention as a physiological foundation, aiming to help novices meditate starting with the body and gradually gain essential meditation skills. A cross-sectional study on 12 novices indicated that the participants reported less mind-wandering during HAM compared with the classic breath-counting meditation (BCM). In a further longitudinal study, the experimental group with 10 novices showed significantly improved performance in several attentional tests after 5 days’ practice of HAM. They tended to show more significant improvements in a few tests than did the control group performing the 5-day BCM practice. To investigate the brain activities related to HAM, we applied functional near-infrared spectroscopy (fNIRS) to record cerebral hemodynamic responses from the prefrontal and sensorimotor cortices when performing HAM, and we assessed the changes in cerebral activation and functional connectivity (FC) after the 5-day HAM practice. The prefrontal and sensorimotor regions demonstrated a uniform activation when performing HAM, and there was a significant increase in the right prefrontal activation after the practice. We also observed significant changes in the FC between the brain regions related to the attention networks. These behavioral and neural findings together provided preliminary evidence for the effectiveness of HAM on attention enhancement in the early stage of meditation learning.

## Introduction

Maintaining attention on a current undertaking and resisting the occurrence of unrelated thoughts are vital aspects of human behaviors. Considerable literature has indicated the broad benefits of intensive meditation practice on attentional performance, mainly including better control over the distribution of limited attentional resources (Van Leeuwen et al., 2009; Slagter et al., 2011), improvement in executive control (Tang et al., 2007; Van den Hurk et al., 2018), and reduced mind-wandering (Mrazek et al., 2013). These findings provide evidence for the claim that meditation benefits the cognitive process, particularly the features of conflict monitoring and attention control (Lutz et al., 2008; Malinowski, 2013; Posner et al., 2015; Tang et al., 2015).

Meditation encompasses a family of practices, and currently, focused attention meditation (FAM) is one of the most widely used and investigated types (Tang and Posner, 2013; Lippelt et al., 2014). FAM is usually the starting point for novice meditators. During this meditation, practitioners are required to maintain attention on specific content such as breathing or a candle flame (Vago and Silbersweig, 2012). In the breath-counting meditation (BCM), for example, practitioners focus on the breathing constantly and count each respiratory cycle silently from 1 to 10 and then start again from 1. Meanwhile, they are instructed to let intervening thoughts pass by and keep concentration on respiration (Levinson et al., 2014; Milz et al., 2014). However, BCM is not as simple as it sounds, because involuntary mind-wandering frequently happens, especially for novices. They often report that they stopped counting, counted over 10, or had to reflect intensively to figure out what was the next count during the BCM practice. Given that mind-wandering is a naturally occurring phenomenon, novices are generally unaware of mind-wandering at the moment it occurs. Consequently, keeping concentration continuously on the breathing is so hard that the sufficient meditative time is short in the early stage of meditation. Besides, practitioners’ attentional states are evaluated solely by themselves, and this limits the ability of meditation coaches to provide a timely reminder when the practitioners’ attention shifts away from their breathing.

Human’s haptic channel is a unique sensory modality in terms of combining sensory perception and motor output. Compared with typical attentional tasks using visual stimuli, haptic-related tasks could invoke internal somatic attention and help participants control the focused spotlight of attention spontaneously. There existed but a few attempts that investigated the effects of somatosensory signals on attention and the possibility of enhancing attention through haptic tasks (Bauer et al., 2006; Dvorkin et al., 2013; Sanchez-Lopez et al., 2014). Our previous work indicated that a 5-day training of force-control tasks with pure haptic feedback could promote short-term attention (Wang et al., 2014). Driven by the beneficial effects of meditation on various attention functions, in this paper, we proposed a *haptics-assisted meditation* (HAM) by incorporating force control and meditation practice. We further hypothesized that this novel paradigm (i) could help novices reduce mind-wandering during the meditation practice and (ii) has the potential in attention enhancement.

To validate the first hypothesis, 12 participants without meditation experience conducted HAM and the traditional BCM for the same duration. Self-reports for the mind-wandering during the two meditation practices were recorded. Furthermore, another 20 novices engaged in a longitudinal experiment aiming to address the second hypothesis. In the experiment, an experimental group and a control group performed a 5-day training of HAM and BCM, respectively. A battery of tests was conducted to assess attentional performance of the two groups before and after the training. In addition, brain hemodynamic responses of the experimental group were measured by functional near-infrared spectroscopy (fNIRS) to explore the possible effects of the short-term HAM training on brain attentional functions.

Functional near-infrared spectroscopy allows for non-invasive monitoring of cerebral concentration changes in oxygenated (oxy-Hb) and deoxygenated (deoxy-Hb) hemoglobin without severe restrictions on body movements and physical environment (Boas et al., 2014). These features make fNIRS well suited for monitoring the neural activity during HAM where natural movements of hands and metallic sensors are necessary. fNIRS has been used to investigate neural activities related to meditation in recent years and indicated a consistent enhancement in the activation of the prefrontal area after meditation practice. The increased activation in the prefrontal cortex (PFC) tended to correlate with better performance in the cognitive tests (Endo et al., 2013; Ji et al., 2019). Meditation practitioners and non-practitioners showed a significant difference in the prefrontal activation during meditation. An increase in oxy-Hb and a decrease in deoxy-Hb were observed in practitioners as compared with non-practitioners (Cheng et al., 2010). Similar hemodynamic differences were also found in the auditory cortex of meditation experts and controls (Gundel et al., 2018). Their findings showed that experts had an increased activation in auditory areas during meditation and also had a more widespread activation pattern beyond the meditative task itself, such as resting state, indicating possible long-term effects on the brain. A longitudinal study further demonstrated that a 6-week practice significantly improved the oxy-Hb at all parts of the PFC in meditation novices as compared with controls who conducted relaxation training for the same duration (Gagrani et al., 2018). For participants with some meditation experience, a 20-min meditation practice increased cerebral oxygenation and enhanced their performance during the Stroop color–word task (Deepeshwar et al., 2015). These findings uniformly suggested an enhanced activation related to meditation practice and provided evidences for the feasibility of fNIRS in meditation research.

With this background, the present study employed fNIRS to assess the effects of the proposed HAM on cerebral hemodynamic responses. Given that the PFC is a crucial structure for cognitive functions, and the sensorimotor cortex (SMC) mainly involves in body sensation and force control, we recorded fNIRS signals from these regions and investigated how HAM affected the cerebral activation in this study. Besides, functional connectivity (FC) is defined as a robust temporal dependency among neural activation patterns, which could indicate coordinated activities between distinct cerebral regions and provide valuable information about the brain communication network (Friston, 2011). For this, we also investigated the FC between the bilateral PFC and SMC to better understand how these regions interacted with each other during HAM.

## Materials and Methods

### Participants

Twelve students without meditation experience from Beihang University participated in experiment 1. They were randomly assigned to group A and B. Group A carried out BCM on the first day and HAM at the same time of the second day, whereas group B carried out HAM on the first day and BCM on the second day. Another 20 naive students from Beihang University who were not involved in experiment 1 were recruited for experiment 2. They were assigned to an experimental or control group randomly with the restriction that gender composition was matched between groups. Experimental participants attended HAM for 5 days for 60 min/day. The controlled participants were given the same number and length of training sessions but attended BCM. There was no significant difference in gender, age, and years of education across groups for both experiments (independent-samples *t*-tests on age and years of education; see Table 1).

**TABLE 1:** Demographics of participants.

	**Experiment 1**			**Experiment 2**		
		
	**Group A**	**Group B**	***p*-values**	**HAM group**	**BCM group**	***p*-values**
Gender	2 F, 4 M	2 F, 4 M	\	4 F, 6 M	4 F, 6 M	\
Mean age (SD)	24.2 (1.7)	23.7 (1.6)	0.62	23.1 (1.7)	23.1 (2.3)	1.00
Mean years of education (SD)	17.7 (1.8)	17.2 (0.8)	0.54	16.5 (1.3)	17.2 (1.9)	0.35

*F denotes females, and M denotes males. Years of education indicate the number of academic years a person completed in a formal program provided by elementary and secondary schools, universities, colleges, or other formal postsecondary institutions.*

All participants in the experiments were right-handed and meditation naive without any previous meditation experience and have normal or corrected-to-normal visual acuity. All of them were confirmed having no history of mental health problems and not receiving any psychopharmacological treatments. Written informed consent was obtained from all participants before the study, and this study was approved by the State Key Laboratory of Virtual Reality Technology and Systems of China.

### Procedures

**FIGURE 1:**
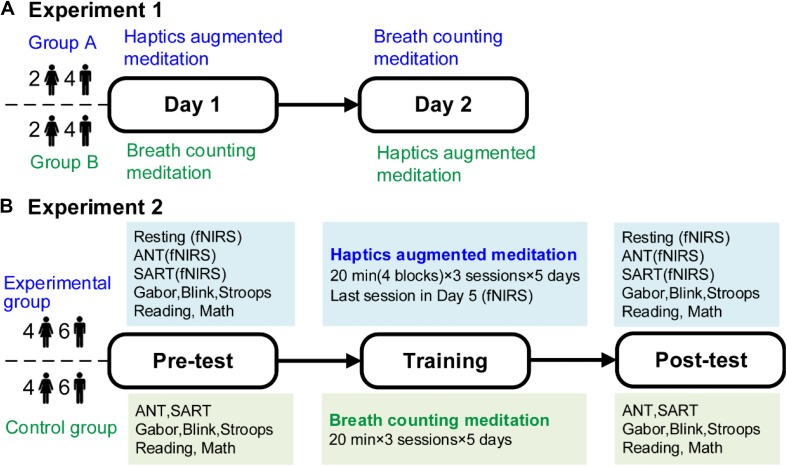
Experimental procedures. **(A)** Experiment 1. Twelve participants carried out HAM and BCM on two consecutive days. **(B)** Experiment 2. Twenty participants were allocated randomly to the experimental or control group who conducted 5-day training of HAM or BCM, respectively. Both groups conducted a battery of tests before and after the training. For the experimental group, fNIRS signals during ANT and SART before and after the training, and during the last training session were recorded. HAM, haptics-assisted meditation; BCM, breath-counting meditation; fNIRS, functional near-infrared spectroscopy; ANT, attention network task; SART, sustained attention to response task.

Figure 1 illustrates the experiment procedures. Prior to formal experiments, participants were provided with written instructions and sufficient practice to make sure they were thoroughly familiar with the requirements. In experiment 1, participants performed the HAM and BCM practices separately at the same time for two consecutive days. Each practice lasted 75 min, split into four sessions of 15 min with 5-min breaks between two adjacent sessions. For each participant, the number of reported mind-wandering episodes during the HAM and BCM practices was recorded separately.

In experiment 2, the experimental and control groups conducted a 5-day training of HAM and BCM, respectively. Both groups performed a 60-min training each day, including three sessions of 20 min with a 5-min break following each session. The two groups were given a battery of tests before training and immediately after the final training session. These tests were arranged in two consecutive days to avoid fatigue. Specifically, the Blink, Stroop, Reading, and Gabor tasks were performed on the first day, and the attention network task (ANT), sustained attention to response task (SART), and Math task were on the second day. There was a 10-min break after each test. Besides, fNIRS measurements were conducted when the experimental participants performed ANT and SART in pretests and posttests. fNIRS signals during the last training session were also recorded from all experimental participants.

#### Haptics-Assisted Meditation

**FIGURE 2:**
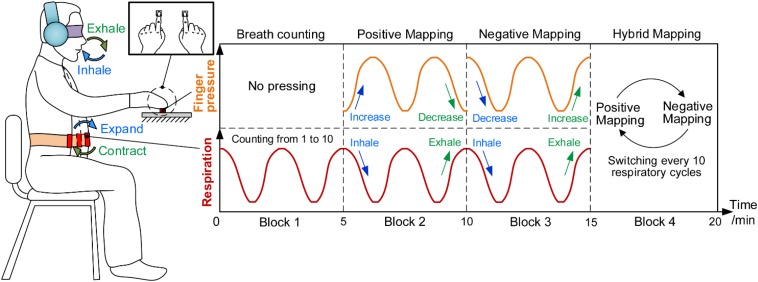
Haptics-assisted meditation. Participants conducted the breath-counting task in block 1. In block 2, “positive mapping” mode, participants were required to increase the fingertip pressure when inhaling and decrease when exhaling. Block 3, “negative mapping” mode, is similar to block 2 with the difference that participants decreased the fingertip pressure when inhaling and increased when exhaling. In block 4, “hybrid mapping” mode, participants performed the positive mapping or negative mapping mode every 10 respiratory cycles.

Figure 2 shows the procedure of HAM during which participants sat in a chair in front of a fixed force-sensor plate and tied a respiration sensor to their abdomen. They also wore an eyeshade for eliminating visual disturbance and a pair of head-mounted earmuffs for eliminating noise. Because deep breathing practice was proved to be beneficial for a variety of cognitive functions and widely used in meditation, participants were instructed to keep a constant, slow, and deep diaphragmatic breathing during the task (Brown and Gerbarg, 2009). More specific requirements for breathing were described in our previous study (Zheng et al., 2019).

Each 20-min session of HAM consisted of four 5-min blocks. During block 1, participants conducted the breath-counting task that is the same as what the control group did. During block 2, “positive mapping,” participants pressed two force sensors using their left and right index fingertips synchronously, increasing the fingertip forces when inhaling and decreasing the forces when exhaling. They were asked to try their best to control the change of fingertip forces to match with their respiratory rhythm. Block 3, “negative mapping,” was similar to block 2 with the difference that participants were required to decrease the forces when inhaling and increase the forces when exhaling. During block 4, “hybrid mapping,” participants performed positive mapping or negative mapping every 10 respiratory cycles and then switched to another mapping mode. The participants, therefore, needed to perform the breath counting simultaneously to ensure the correct switch between the two mapping modes. At the end of each block, participants would hear a short music to remind them to enter into the next block.

It should be noted that a simplified HAM was performed in experiment 1. While pressing with bilateral index fingers in experiment 2, participants were only required to press one force sensor using their right index fingers in the simplified task and thus were allowed to report mind-wandering using left hands conveniently. During experiment 1, participants performed two sessions of positive mapping and two sessions of negative mapping in the sequence of positive, negative, positive, and negative. They were instructed to immediately report whenever they became aware of mind-wandering by clicking a digital counter (GSQ100, Paulone Inc., China) in their left hands and then refocused on the task. Mind-wandering here was defined as “forgetting to press the sensor when breathing in or out,” “performing the wrong mapping mode,” or “being aware that thoughts had drifted away from breathing or pressing.” These guidelines were instructed to all participants before the experiment. Besides, participants were not required to report mind-wandering in experiment 2.

Data of respiration and fingertip force were recorded at a sample rate of 200 Hz. A respiration sensor belt (used in combination with NeXus-10 Mark II, Mindmedia Inc., Netherlands) was tied to the participant’s abdomen to record respiratory signals. Two force sensors (FSG15N1A, Honeywell Inc., United States) were mounted on a fixed plate to measure the fingertip pressures exerted by two index fingers.

#### Breath-Counting Meditation

During BCM, participants sat in a spacious and silent room and held a digital counter in their left hands. They were instructed to sit on a chair comfortably, close eyes, and silently count each respiratory cycle from 1 to 10 and then repeat from 1. The requirements for the breathing here were consistent with the requirements in HAM. The participants were also asked to concentrate entirely on the task and press the counter immediately to report the mind-wandering whenever they became aware of losing track of the task. Specifically, mind-wandering here refers to “stopping counting,” “counting over 10,” or “had to reflect intensively to figure out what was the next count.” Following the report, participants should bring the focus back to the breathing and count again from 1.

When performing BCM, participants followed a compact disc with body posture adjustment and relaxed practice accompanied by a music background. Instructions on the compact disc were from standard guided audio recordings. A coach observed facial and body cues to identify those who were struggling with the practice and gave proper instructions immediately after the training session. At the end of each session, the counters’ number of all participants was recorded and reset to zero. The number of subjective mind-wandering reports was recorded using digital counters (GSQ100, Paulone Inc., China) from all participants.

#### Test Tasks

Five computerized attentional tasks (ANT, SART, Blink, Gabor, and Stroop) and two real-world tasks (Reading and Math) were used to assess the changes of attentional performance from pretests to posttests. We increased the difficulty of these tasks because all participants in this study were healthy graduate students. Thus, some parameters of these tasks such as the number of trials, duration of stimulus, and time limit for response were modified based on the parameters used in previous studies.

Attention network task has been used to assess the effects of meditation on three basic aspects of attention including alerting, orienting, and executive control (Fan et al., 2002; Tang et al., 2007). In each trial of the task, first, there was a fixation cross at the center of the screen for 500 ms. Then, a cue was presented for 100 ms to inform the target would occur right now and to provide information on where the target would be. Following the cue, there was a fixation period for 300 ms, and then the target and flankers appeared simultaneously. Participants needed to respond to the direction (left or right) of the arrow target by clicking the corresponding buttons on the keyboard as fast and accurately as possible. The arrow target was surrounded by flankers that pointed in either the same or opposite direction. The target and flankers were presented until the participant responded, but for no longer than 1,000 ms. After participants made a response, the target and flankers disappeared immediately, and there would be a short resting period for 1,500 ms. Following this period, participants received the next trial until completing 108 trials. The whole task lasted for about 6 min. The average accuracy and response time (RT) were also calculated to assess the overall performance of the task. Subtraction of RTs was used to obtain scores for the performance in alerting, orienting, and executive control, as described by Fan et al. (2002).

Sustained attention to response task is a go/no-go continuous performance task that requires participants to respond to the go stimuli as quickly as possible, and no response to no-go stimulus appears infrequently (Helton et al., 2009; Levinson et al., 2014). In this task, participants received 240 trials corresponding to 240 single digits (24 each from 0 to 9). In each trial, a random digit was presented for 500 ms at the center of the computer screen and followed by a fixation cross for 500 ms. Participants were instructed to respond to each digit via a key press as fast as possible when the digit appeared but to withhold their response when that digit was a 3. The digit would disappear, and the next digit would appear no matter the participant responded or not. The duration from digit to digit lasted for 1,000 ms, and thus, the whole task took 4 min. The RT and accuracy were calculated for all trials. The accuracy indicated the whole proportion of trials successfully responding to go stimuli and not responding to no-go stimuli.

Attentional blink task (Blink) is a classic test to assess the level of the attentional-blink deficit (Van Leeuwen et al., 2009). The deficit indicates that when two targets (T1 and T2) embedded in a rapid stream of events are presented in close temporal proximity, the second target is often not seen and is believed to result from competition between the two targets for limited attentional resources. A recent study has found a smaller attentional blink and reduced brain-resource allocation to the first target after 3 months of meditation training (Slagter et al., 2007). In this study, we also used this task to assess the possible effects of HAM on the distribution of attentional resources. Each trial started with a 1,780-ms fixation cross, followed by a rapid serial stream of 15 or 19 letters. The letters were from the alphabet except B, I, O, Q, and S, because these letters are likely to be confused with numbers. Each letter was presented randomly for 25 ms, followed by a 34-ms blank. For each trial, one or two letters were replaced by a random number between 2 and 9. When only one letter was replaced by a number, a second letter was replaced with a blank screen (T2-absent trial). There could be short (336 ms) or long (672 ms) interval between the first (T1, a number) and second target (T2, a number or the blank screen). Participants were asked to report T1 and T2 by typing the numbers in order on a keyboard after the stream ended. If participants were sure no T2 was presented, they entered zero for this number, whereas they had to guess T2 if they thought T2 had been presented but could not ensure which number it was. They were informed to report as accurately as possible, and there was no time limit for response. The next trial began after the second response. Participants performed 102 trials of the task, consisting of 48 short-interval/T2-present trials, 18 long-interval/T2-present trials, 18 short-interval/T2-absent trials, and 18 long-interval/T2-absent trials with a random order. The proportion of the correct trials (T1 and T2 were both reported correctly) was calculated as task accuracy.

Stroop words task has been used to investigate the effects of meditation practice on executive functions and has revealed significant differences between meditators and non-meditators (Moore and Malinowski, 2009; Moore et al., 2012). Stimuli in the task were the four words RED, BLUE, GREEN, and YELLOW, presented in the same color as the written word in congruent trials (e.g., RED presented in red) or in different colors in incongruent trials (e.g., RED presented in blue). Participants were instructed to indicate the color of each word while ignoring the semantic meaning of the word. These words in the task were presented in Chinese characters, given that all participants engaging in this study are Chinese. In each trial, a fixation cross was first presented at the center of the screen for 500 ms, followed by the color word that was presented for 1,000 ms. Participants were required to enter their responses by pressing the corresponding keys as fast and accurately as possible when the word appeared. Four keys on the keyboard were color coded and used to provide comfort for the participant when responding, including the keys “a” (red, left middle finger), “.” (yellow, left index finger), “x” (green, right index finger), and “ ’ ” (blue, right middle finger). The whole test consisted of 96 trials that included 64 incongruent trials and 32 congruent trials. These trials were presented in a random order. Each incongruent stimulus appeared in each of the three other colors with equal frequency. After the test, the accuracy for all trials and the RT for incongruent trials were calculated for each participant.

**FIGURE 3:**
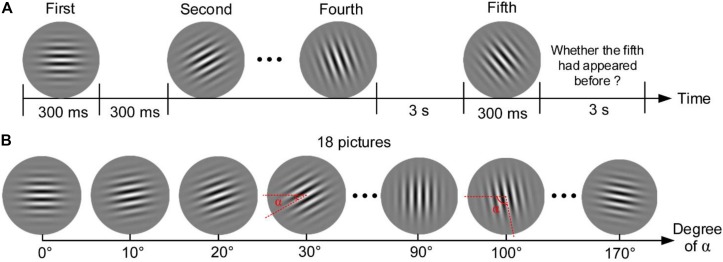
Gabor patch discrimination task. **(A)** Procedure of one trial. **(B)** Eighteen pictures that could be presented in the task.

The Gabor patch discrimination task (Gabor) in the present study was modified from a task in the study of Wang et al. (2014). The task consisted of 100 trials. In each trial, four pictures with different angles of stripes were presented on the center of the computer screen for 300 ms, and each picture was followed by a 300-ms blank (see Figure 3). The fifth picture was then displayed for 300 ms after a 3-s blank. Participants were provided 3 s to report whether the final picture had appeared in the previous four pictures via key pressing. The next trial began after the response. There could be 18 pictures with a minimum angle difference of 10° in this task, as shown in Figure 3B. The five pictures presented in each trial were assigned randomly from the 18 pictures with a restriction that the minimum angle difference across the five pictures was not less than 30°. For example, the pictures of 0°, 20°, and 30° would not appear in the following four pictures if the picture of 10° had been presented first. This restriction aimed to ensure clear discrimination for the presented pictures for all participants in each trial. Accuracy of the task was calculated to assess the performance of sustained attention and working memory.

The five tests mentioned above were all presented on a 21-in. cathode-ray tube (CRT) monitor (100-Hz refresh rate, 1,024 × 768 resolution) and were developed in the C programing environment. The fixation cross and stimulus in these tasks always appeared centrally on the screen. Before each test, participants performed 20 practice trials at least to get familiar with the test.

We also conducted two non-computerized but more real-world tasks (Math computation and Reading task as follows) to assess the possible transfer effect of HAM on attention enhancement to activities in real life. A similar math computation task has been used in our previous study and revealed significant enhancement of performance after a 5-day training of force-control tasks (Wang et al., 2014). In this test, 32 lines of Arabic numbers (the font of Times New Roman, size 16 pt) were printed on a piece of A4 paper, and each line included 30 numbers with a range of 0–9. Participants were required to mark the pairs of two adjacent numbers whose sum equaled to 10 as many as possible within the time limit of 8 min. They were informed to mark the numbers line by line with a pen and were not allowed to go back to previous lines to change their answers. The score of the test was computed manually by subtracting the number of wrongly marked pairs from the number of correctly marked pairs. In addition, the sequences of digits were modified for the posttest, but the total number of pairs summing to 10 was the same as the pretest.

In the Reading task, four passages were selected from the texts with the same difficulty level in the Nelson-Denny Reading Comprehension Tests (Feng et al., 2013). These passages were all related to history with similar numbers of words (approximately 450 words) and were modified to develop an easier version considering all participants are not native speakers of English. The modification was done by translating and marking difficult or low-frequency English words with Chinese. The four passages were then assigned to two sets. The two sets were tested as the pretest and posttest with a balanced order across participants. Ten single-choice comprehension questions and 20 misspelled words were designed for each set. The questions were closely related to the content of the passages. The misspelled word was done by exchanging the order of two adjacent letters, but the meaning of the word was not easy to be misunderstood; for example, “young” was written as “yuong.” Participants were required to answer the questions, mark the misspelled words they found with circles, and mark “/” in the position they were reading whenever they became aware of mind-wandering. Participants would be stopped when the test duration exceeded 40 min. The task score was computed as S1 + S2 − S3 − S4 with the presence of four components: number of right answers of the single-choice comprehension questions (S1), number of the misspelled words that were found accurately (S2), number of right-spelled words but marked as misspelled (S3), and number of mind-wandering self-reports (S4).

#### Temporal Synchronization

**FIGURE 4:**
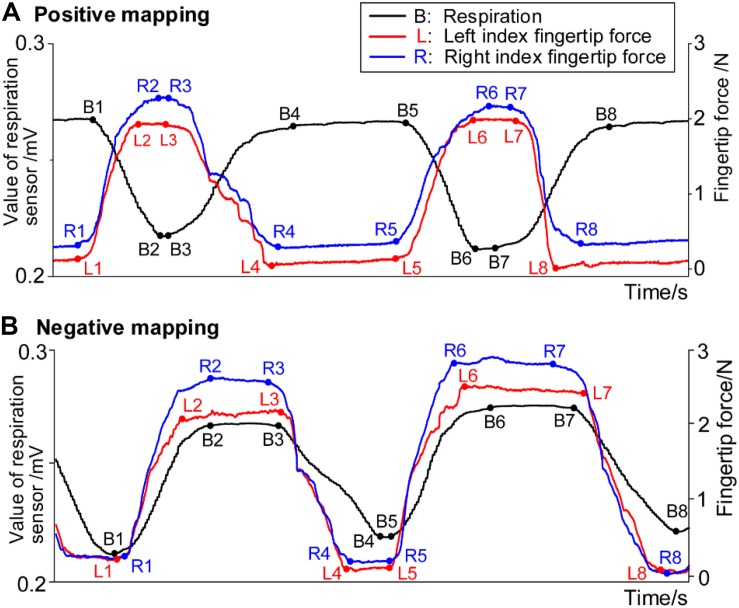
Example data of respiration signals and forces from bilateral index fingers in **(A)** positive mapping and **(B)** negative mapping.

Figure 4 shows the example data of respiration and fingertip forces during the HAM practice. Descent of the black curve in the figure represents inhalation, and the curve’s ascent represents exhalation. The rise of the red and blue curves represents the increase of the left and right index fingertip forces, respectively. Similarly, the going down of the curves represents the decrease of the forces.

The temporal synchronization (Δ_TS_) was defined as the time difference between the moment that respiration signals and fingertip forces begin to change. Δ_TS_ for the left and right hands was computed separately, considering that there could exist a difference between the dominant and non-dominant hands. Four Δ_TS_ values can be computed in one respiratory cycle as listed in Table 2. With Δ_TS_ in block 2 taken as an example, the four Δ_TS_ values include the time difference (i) between the moment of beginning to inhale and beginning to increase the fingertip forces, (ii) between the moment of inhalation end and the moment of the forces reaching the maximum, (iii) between the moment of beginning to exhale and beginning to decrease the forces, and (iv) between the moment of exhalation end and the moment of the forces reaching the minimum. The B1, B2, B3, and B4 denote the data points of beginning to inhale, inhalation ending, beginning to exhale, and exhalation ending, respectively. The L1, L2, L3, and L4 (R1, R2, R3, and R4) denote the data points of left (right) index fingertip force beginning to increase, reaching a maximum, beginning to decrease, and reaching a minimum, respectively. Considering that the respiratory and fingertip force signals reached a plateau near the peak and valley, calculating those start and end data points of the plateaus was the key to the algorithm for computing Δ_TS_.

**TABLE 2:** Definition of temporal synchronization in positive and negative mapping.

**Mode**	**Definition of Δ_TS_ (time difference between the moment of event A and B)**		**Calculation of Δ_TS_**	
		
	**Event A**	**Event B**	**Left hand**	**Right hand**
Positive mapping	Starting to inhale	Starting to increase force	∣*T*(B1) − *T*(L1)∣	∣*T*(B1) − *T*(R1)∣
	Inhalation end	Force reaching the maximum	∣*T*(B2) − *T*(L2)∣	∣*T*(B2) − *T*(R2)∣
	Starting to exhale	Starting to decrease force	∣*T*(B3) − *T*(L3)∣	∣*T*(B3) − *T*(R3)∣
	Exhalation end	Force reaching the minimum	∣*T*(B4) − *T*(L4)∣	∣*T*(B4) − *T*(R4)∣
Negative mapping	Starting to exhale	Starting to increase force	∣*T*(B1) − *T*(L1)∣	∣*T*(B1) − *T*(R1)∣
	Exhalation end	Force reaching the maximum	∣*T*(B2) − *T*(L2)∣	∣*T*(B2) − *T*(R2)∣
	Starting to inhale	Starting to decrease force	∣*T*(B3) − *T*(L3)∣	∣*T*(B3) − *T*(R3)∣
	Inhalation end	Force reaching the minimum	∣*T*(B4) − *T*(L4)∣	∣*T*(B4) − *T*(R4)∣

We developed the algorithm in MATLAB (2018b, MathWorks, Inc., United States) and stated the steps as follows. First, a bandpass filter (0.5–100 Hz) and moving average filtering were used to remove apparent noise. Second, we segmented the filtered dataset into segments of each respiratory cycle by setting the minimum interval among two adjacent peaks and the minimum amplitude of peaks. So the moments of the maximum points and minimum points in each data segment can be obtained. Last, for each data segment, the least square method was used to obtain a straight line that passed the maximum or minimum point to fit the plateaus of peak or valley. The intersections of this straight line with the filtered signals were then calculated. Generally, more than one intersection would be obtained. The intersection with the minimum abscissa (i.e., the corresponding moment) was the start point of the peak or valley plateau, and the intersection with the maximum abscissa was the end of the plateau.

#### Near-Infrared Spectroscopy

**FIGURE 5:**
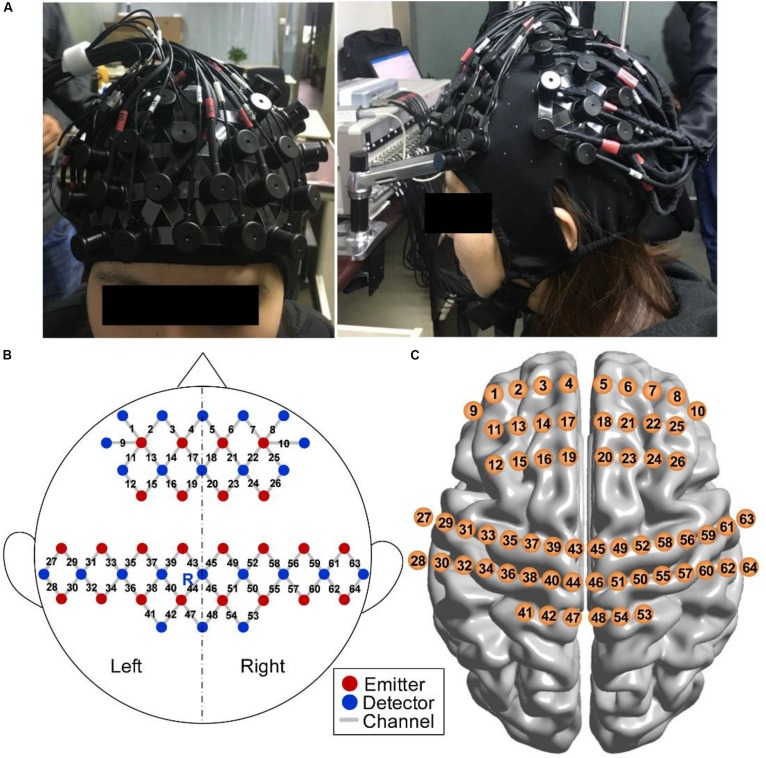
fNIRS measurement. **(A)** Photograph of fNIRS measurement on a participant. **(B)** Schematic of imaging pad. Twenty-two emitters (red dots) and 24 detectors (blue dots) were symmetrically placed on the bilateral hemispheres and constituted 64 measurement channels (gray lines), allowing for the prefrontal and sensorimotor cortices to be measured. The emitter R was placed in Cz of the international 10–20 system. **(C)** Anatomical position of each measurement channel.

An fNIRS system (NirScan, DanYang HuiChuang Medical Equipment Inc., China) with three wavelengths (740, 808, and 850 nm) of near-infrared light was used to record cerebral hemodynamic changes with a sampling rate of 17 Hz. Twenty-two emitters and 24 detector probes were plugged into a soft cap with an inter-optode distance of 30 mm. The measurement channels were located midway between each emitter–detector pair. A total of 64 channels were symmetrically distributed in bilateral hemispheres and were positioned over the PFC and SMC in accordance with the international 10–20 electrode placement system (Jasper, 1958). The emitter R marked in Figure 5 was placed in Cz.

We measured fNIRS signals from all participants when they performed ANT and SART in the pretest and posttest and when they conducted the last session of HAM practice. We also recorded 5 min of fNIRS signals during resting state before each fNIRS measurement. For the fNIRS data, variables of interest were relative changes in the concentration of oxy-Hb and deoxy-Hb compared with that at baseline. The baseline was defined as 1 min at rest before the ANT and SART, as well as before each block of HAM. We should note that there was no 1-min rest before each block for those HAM sessions without fNIRS measurements. During the resting state and baseline periods, participants were instructed just to rest and relax without a specific focus.

#### Data Analysis and Statistics

All analyses for behavioral and fNIRS data were completed by MATLAB (2016b, MathWorks, Inc., United States). For the self-reports in experiment 1, we conducted paired *t*-tests to compare the number of mind-wandering reported in HAM and BCM. We also used paired *t*-tests to compare the performance in the pretests and posttests within the HAM or BCM group in experiment 2. In order to assess the difference in the performance changes between the two groups, we first calculated the change of performance by subtracting the performance in pretests from the performance in posttests for each group, and then we compared the performance changes between the two groups by independent-samples *t*-tests.

For fNIRS data, the preprocessing was conducted using the open-source software HOMER2^1^ implemented in MATLAB. All recorded signals were first processed by the principal component analysis approach to remove signal contamination, such as systematic artifacts (Tak and Ye, 2014). The motion artifact segments greater than five standard deviations from the mean were detected and replaced with their spline interpolation on the basis of neighboring signals. The corrected signals were then bandpass filtered between 0.01 and 0.2 Hz to remove baseline drift and physiological noises. The filtered optical data were finally converted into hemoglobin signals in accordance with the modified Beer–Lambert law (Cope and Delpy, 1988).

Data of oxy-Hb and deoxy-Hb were individually averaged according to the task condition (ANT_pre, ANT_post, SART_pre, SART_post, and Block1, 2, 3, and 4 in HAM) for each channel. The first 60 s of all these conditions was discarded from the analysis to give participants enough time to reach a steady state. The analysis for the HAM period was performed by the block, resulting in a 240-s average for each participant and each block. The fNIRS analyses for the conditions of ANT_pre, ANT_post, SART_pre, and SART_post were performed by task excluding wrong trials (i.e., only including all correct trials). Then, the values of oxy-Hb and deoxy-Hb were converted into *z*-scores by a Fisher *z*-statistics before statistical tests.

We further analyzed FC using the Pearson correlation coefficient and a seed-based approach (Tak and Ye, 2014). The hemoglobin data during the task period (discarding the first 60 s as described above) were used to compute the Pearson correlation coefficients between the seed channel and all the other channels, generating a 64 × 64 *r*-value correlation matrix for each participant and each condition. These individual-level correlation coefficients were then converted to *z*-values via Fisher’s *r*-to-*z* transformation to improve normality, resulting in eight 64 × 64 *z*-value FC matrices corresponding to the eight conditions for each participant. To perform a group-averaged FC comparison, we first averaged these individual *z*-value FC matrices within each condition and then converted the group-level *z*-value matrix into an *r*-value matrix via Fisher’s *z*-to-*r* transformation for each condition. For reasons of conciseness, only the FC analysis of oxy-Hb data was reported in this study, although the deoxy-Hb data had gone through the same analysis.

Statistical analyses were applied to the values of oxy-Hb, deoxy-Hb, and the FC matrices separately in a channel-wise manner. Paired *t*-tests were conducted for each channel to test whether there were significant differences before and after the HAM training (*p* < 0.05). We also performed paired *t*-tests for each channel to identify the channels showing significant changes in each block of HAM as compared with those during the resting state. Here, the method of false discovery rate (FDR) was applied to each channel to control the family-wise error rate (Singh and Dan, 2006). Significance values reported in this study were those that survived the FDR.

## Results

### Behavioral Results

#### Performance Changes After Haptics-Assisted Meditation and Breath-Counting Meditation Training

**FIGURE 6:**
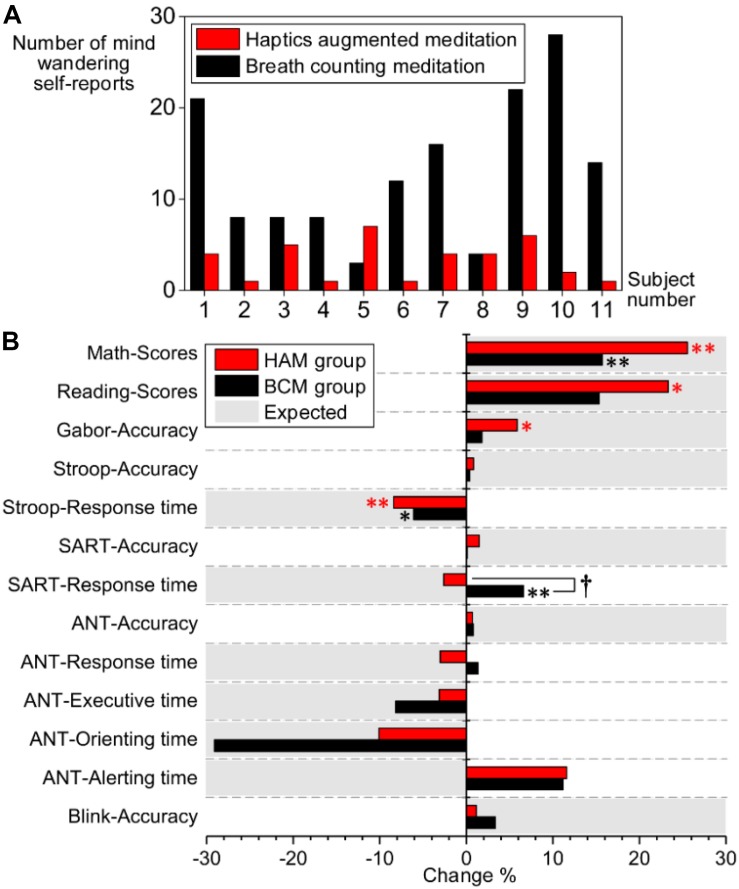
**(A)** Number of mind-wandering self-reports during HAM (red bars) and BCM (black bars). **(B)** Comparison of performance changes between the HAM (red bars) and BCM groups (black bars). The vertical axis indicates the task performance. The horizontal axis indicates the percentage of performance change after training [i.e., (Post – Pre)/Pre × 100%]. The positive values marked in gray represent that increased performance is expected for the task; the negative values marked in gray represent that decreased performance is expected. ^∗^ and ^∗∗^ denote significant changes of performance within the HAM or BCM group; ^∗^, *p* < 0.05; ^∗∗^, *p* < 0.01. †denotes a significant difference in the performance changes between the HAM and BCM groups (*p* < 0.05). HAM, haptics-assisted meditation; BCM, breath-counting meditation.

Figure 6A presents the number of mind-wandering self-reports in experiment 1. The paired *t*-test indicated significantly less mind-wandering (*p* = 0.003) in HAM (1–7, mean 3.27 ± 2.195) than in BCM (3–28, mean 13.09 ± 7.981). One participant was excluded from the analysis because he reported drowsiness during the experiment.

For experiment 2, we compared the attentional performance in the pretests and posttests using paired *t*-tests. After the HAM training, significant improvements were observed in the accuracy of the Gabor test (*p* = 0.034), and in the scores of Reading (*p* = 0.031) and Math tests (*p* = 0.002), as shown in Table 3. The performance of the Stroop task also improved significantly (*p* = 0.003), indicated by a decrease in the mean RT. Besides, the accuracy in Blink, ANT, SART, and Stroop also increased, although these improvements were statistically insignificant. The mean RT of SART and the orienting time, executive time, and mean RT of ANT were slightly shorter than those in the pretests. For the BCM group, we found significantly improved performance in Math (higher scores, *p* = 0.006) and Stroop (shorter RT, *p* = 0.028). However, the mean RT of SART became longer (*p* = 0.006) after the training.

**TABLE 3:** Performance of HAM group in pretoup separately and then compasests and posttests.

**Tasks**	**Index**	**Pre (mean ± SD)**	**Post (mean ± SD)**	**Change %**	***t*-value**	***p*-value**
Blink	Accuracy	0.86 ± 0.67	0.87 ± 0.08	1.16	0.515	0.619
ANT	Alerting time (ms)	36.89 ± 17.32	41.16 ± 15.87	11.58	0.895	0.394
	Orienting time (ms)	21.87 ± 12.91	19.66 ± 9.37	–10.11	–0.35	0.734
	Executive time (ms)	64.06 ± 19.43	62.05 ± 14.10	–3.13	–0.308	0.765
	Response time (ms)	510.58 ± 32.94	495.14 ± 28.00	–3.02	–1.814	0.103
	Accuracy	0.98 ± 0.02	0.99 ± 0.01	0.71	1.397	0.196
SART	Response time (ms)	308.55 ± 39.30	300.45 ± 29.24	–2.62	–1.126	0.289
	Accuracy	0.95 ± 0.05	0.96 ± 0.03	1.48	1.155	0.278
Stroop	Response time (ms)	820.12 ± 98.24	751.25 ± 101.44	–8.40	–3.927	0.003^∗∗^
	Accuracy	0.97 ± 0.02	0.98 ± 0.02	0.83	1.000	0.343
Gabor	Accuracy	0.65 ± 0.06	0.69 ± 0.07	5.87	2.493	0.034^∗^
Reading	Scores	19.30 ± 6.20	23.80 ± 4.21	23.32	2.558	0.031^∗^
Math	Scores	162.40 ± 27.76	203.90 ± 34.13	25.55	4.432	0.002^∗∗^

*Change = (Post − Pre)/Pre × 100%. ^∗^*p* < 0.05. ^∗∗^*p* < 0.01. ANT, attention network task; SART, sustained attention to response task.*

Moreover, we computed the changes of performance for HAM and BCM group separately and then compared the difference in the changes between the two groups by independent-samples *t*-tests (see Figure 6B). A significant difference was observed only in the mean RT of SART (2.62% decrease in HAM group and 6.60% increase in BCM group, *p* = 0.006). Nevertheless, for HAM group, we found (i) greater improvements in the accuracy of SART (*p* = 0.334), Stroop (*p* = 0.722), and Gabor (*p* = 0.332); (ii) greater improvements in the scores of Math (*p* = 0.180) and Reading (*p* = 0.553); and (iii) greater decrease in the mean RT of Stroop (*p* = 0.414) and ANT (*p* = 0.104) than those for BCM group. These findings suggested that the HAM group showed greater improvements in several tests than did the BCM group, although none of these improvements were statistically significant.

#### Behavioral Performance During Haptics-Assisted Meditation Training

**FIGURE 7:**
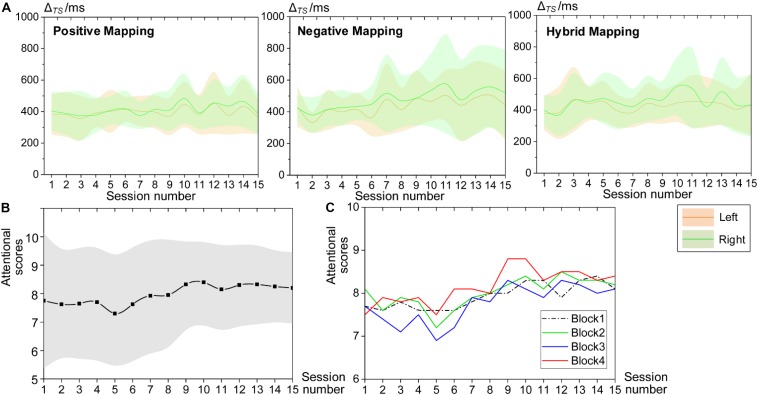
Behavioral performance during HAM training. **(A)** Mean Δ_TS_ values of all sessions for block 2 (positive mapping), block 3 (negative mapping), and block 4 (hybrid mapping). The green patterns represent Δ_TS_ computed from the right, and the yellow patterns represent Δ_TS_ from the left. **(B)** Mean attentional scores for the whole session reported by all participants. **(C)** Mean attentional scores for each block. HAM, haptics-assisted meditation.

We used the temporal synchronization (Δ_TS_) to assess the attentional performance during HAM. The decrease in Δ_TS_ indicates improved performance. Figure 7A shows the mean Δ_TS_ values in blocks 2, 3, and 4 from all sessions. Because all participants engaged in this study were right-handed, and there could exist a difference in Δ_TS_ between the dominant and non-dominant hands, we computed the Δ_TS_ for the left and right hands separately. The mean Δ_TS_ derived from the left and right hands both remained around 400 ms, and there was no significant difference between the bilateral hands, although the Δ_TS_ performed by the left hand seemed to be more stable. Besides, we did not find a significant difference of Δ_TS_ across the three blocks, but the variability of Δ_TS_ across subjects in block 2 (positive mapping) tended to be smaller than that in the other two blocks (negative and hybrid mapping).

Furthermore, participants evaluated their attentional level using a score of 1–10, with 10 being the highest level of attention at the end of each HAM session. Figures 7B,C present the average attentional scores for the whole session and each block, respectively. The subjective attentional scores for the session and the block both showed a trend of increase as the practice sessions increased. The variability of the scores for the whole session between participants also tended to be smaller over time. These findings suggested that HAM enhanced sustained attention ability from subjective judgment.

### Neural Results

#### Hemodynamic Changes After Haptics-Assisted Meditation Training

**FIGURE 8:**
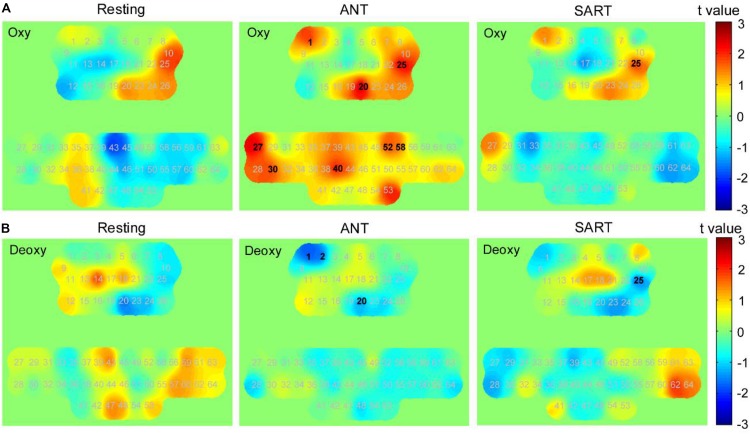
Changes in the concentration of oxy-Hb and deoxy-Hb after training (post minus pre). *t*-value maps obtained by paired *t*-tests on **(A)** oxy-Hb and **(B)** deoxy-Hb. Each number in these maps denotes the numbering of optical channels. The numbers marked in bold denote that the changes in these channels were significant statistically. The positive *t*-values in the color bar represent higher concentration in posttests than in pretests, and the negative values represent lower concentration than in the pretests.

Mean concentrations of oxy-Hb and deoxy-Hb when performing ANT and SART in the posttests compared with the pretests by paired *t*-tests with FDR thresholding. Figure 8 presents a visual depiction of changes in oxy-Hb and deoxy-Hb concerning various cortical regions. Data from oxy-Hb showed a significant increase in the post-ANT in several channels over the PFC (channels 1, 20, and 25; *p* = 0.021, 0.003, and 0.008, respectively), as well as in the left SMC (channels 27, 30, and 40; *p* = 0.009, 0.032, and 0.017, respectively) and the right SMC (channels 52 and 58; *p* = 0.026 and 0.015, respectively) when compared with the pre-ANT. For the deoxy-Hb, we observed a significant decrease in channels 1, 2, and 20 (*p* = 0.032, 0.039, and 0.040, respectively) over the PFC in the post-ANT. Besides, there was a significant increase in oxy-Hb over the right PFC (channel 25, *p* = 0.031) in the post-SART. A significant decrease in deoxy-Hb was also observed in this channel (*p* = 0.022) when compared with the pre-SART. Additionally, after the HAM training, we also found an increase in oxy-Hb and a decrease in deoxy-Hb over the right PFC during the resting state. However, no significant changes were observed following thresholding by FDR.

**FIGURE 9:**
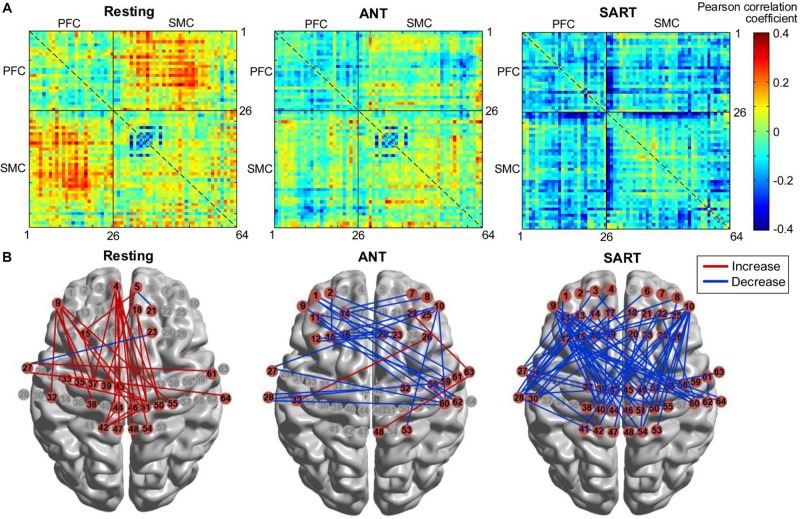
Changes in FC after training (post minus pre). **(A)** FC matrices across all channels. Each number (1–64) in the *x*- or *y*-axis denotes the numbering of optical channels. The color bar indicates the value of the Pearson correlation coefficient. **(B)** FC that increased or decreased significantly (*p* < 0.05) after the training, marked with red or blue lines, respectively. FC, functional connectivity.

Furthermore, the changes in FC matrices across all channels after the HAM training are shown in Figure 9A. Each pixel value in the 64 × 64 correlation matrix corresponds to the value of the change in the group-average Pearson correlation coefficients for oxy-Hb. Paired *t*-tests with the FDR adjustments indicated that there was a significant increase in FC between several channels of the medial PFC and the SMC during resting state. In the posttest, a significantly decreased FC between the left PFC and the right SMC was observed during ANT as compared with the pre-ANT. There was also a uniformly decreased FC between the PFC and SMC in the post-SART. These channel–pairs that increased or decreased significantly (*p* < 0.05) after the training are presented in Figure 9B with red or blue lines, respectively.

#### Hemodynamic Responses During Haptics-Assisted Meditation Sessions

We performed paired *t*-tests with FDR correction for each channel to identify the channels that changed significantly in each block of HAM when compared with those at its baseline (resting state before the block). In block 1, data from oxy-Hb suggested a trend of increase in most parts of the PFC and SMC. The results from paired *t*-tests showed a significant increase in channels 12 and 26 (*p* = 0.046 and 0.043); however, the significant values did not survive after the FDR thresholding. The deoxy-Hb data also demonstrated a trend of decrease in part of the PFC and the medial SMC, although there was no statistical significance.

Although a slight increase in oxy-Hb and a decrease in deoxy-Hb were observed in block 1, we found a uniform and widespread activation pattern when participants performed blocks 2, 3, and 4, indicated by increased oxy-Hb and decreased deoxy-Hb in most regions of the bilateral PFC and SMC. During block 2, participants showed a significant increase in oxy-Hb in channel 26 located in the right PFC and a decrease in deoxy-Hb in this channel. We also observed the decreased deoxy-Hb in the left PFC (channels 1 and 9). Over the SMC, there was significantly increased oxy-Hb in channels 30, 40, 41, 53, and 55 and decreased deoxy-Hb in channels 30, 40, 51, and 53. For block 3, we observed a similar but more widespread activation in the PFC relative to block 2, including significantly increased oxy-Hb in channels 9, 12, 25, and 26 and decreased deoxy-Hb in channels 1, 7, 9, and 26. There was also a significant increase in oxy-Hb in the medial SMC (channels 40, 41, 46, 47, 53, and 54) and the right SMC (channels 63 and 64), as well as a significant decrease in deoxy-Hb in channels 27, 51, and 53.

**FIGURE 10:**
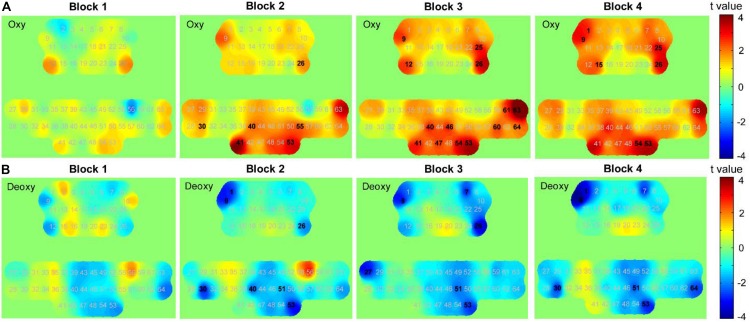
*t*-value maps obtained by paired *t*-tests on **(A)** oxy-Hb and **(B)** deoxy-Hb for each block. The numbers marked in bold denote that these channels showed significant changes than during resting state.

When performing block 4, participants also showed significantly increased oxy-Hb in the PFC including channels 1, 9, 15, 25, and 26. Although there was also a widespread increase in oxy-Hb over most regions of SMC, only the channels 53 and 54 showed a statistically significant increase following the FDR thresholding. The deoxy-Hb data demonstrated a significant decrease in channels 1 and 9 located in the left PFC, as well as in channels 30, 51, 53, and 64 located in the SMC. Figure 10 presents the *t*-value maps of oxy-Hb and deoxy-Hb for each block, and Table 4 summarizes these statistical results.

**TABLE 4:** Summary of the *t*-test results of oxy-Hb and deoxy-Hb.

**Comparison**	**Variable**	**Brain region**	**Channel number**	***p*-values**
Block 2 – Resting	Oxy-Hb	PFC	26	0.021
		SMC	30, 40, 41, 53, 55	0.023, 0.015, 0.002, 0.006, 0.013
	Deoxy-Hb	PFC	1, 9, 26	0.010, 0.008, 0.027
		SMC	30, 40, 51, 53	0.013, 0.007, 0.020, 0.033
Block 3 – Resting	Oxy-Hb	PFC	9, 12, 25, 26	0.028, 0.015, 0.008, 0.012
		SMC	40, 41, 46, 47, 53, 54, 63, 64	0.002, 0.017, 0.014, 0.004, 0.003, 0.001, 0.023
	Deoxy-Hb	PFC	1, 7, 9, 26	0.027, 0.005, 0.007, 0.003
		SMC	27, 51, 53	0.003, 0.03, 0.02
Block 4 – Resting	Oxy-Hb	PFC	1, 9, 15, 25, 26	0.011, 0.015, 0.019, 0.021, 0.017
		SMC	53, 54	0.023, 0.013
	Deoxy-Hb	PFC	1, 9	0.007, 0.007
		SMC	30, 51, 53, 64	0.013, 0.023, 0.030, 0.023

*PFC, prefrontal cortex; SMC, sensorimotor cortex.*

**FIGURE 11:**
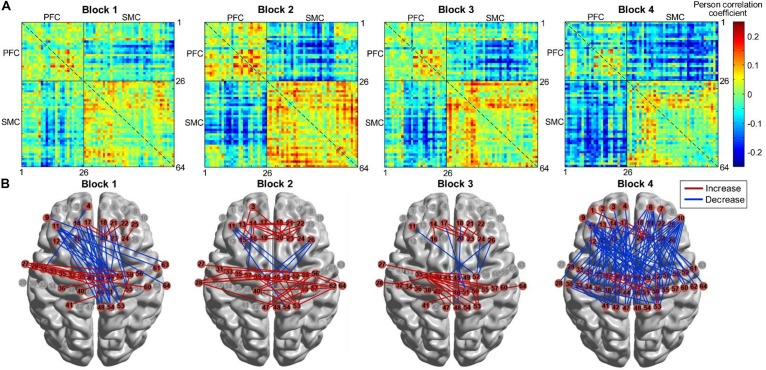
FC for four HAM blocks. **(A)** FC matrices across all channels. **(B)** FC that increased or decreased significantly (*p* < 0.05) marked by red or blue lines, respectively. FC, functional connectivity; HAM, haptics-assisted meditation.

We further compared the differences in FC between each block and its baseline. Figure 11A shows the group-level FC across all channels for each block, and Figure 11B marks those channel–pairs that significantly increased or decreased compared with those at baseline (paired *t*-tests with FDR thresholding, *p* < 0.05) by red or blue lines, respectively. When performing block 1, participants showed a significant decrease in the FC between the PFC (left PFC in particular) and the right SMC. Block 2 and block 3 demonstrated a similar finding that FC within the PFC and the SMC became stronger than during resting state, and only several channels showed weaker coupling between the PFC and SMC. For block 4, although a few channels within the PFC and SMC demonstrated enhanced connectivity, FC between most channels in the PFC and SMC tended to decrease when performing the task.

## Discussion

The present study proposed and implemented a HAM paradigm for attention enhancement. A series of behavioral performance and fNIRS findings suggested an improvement of the proposed paradigm as compared with a classical BCM paradigm. In terms of the behavioral performance, participants reported less mind-wandering in HAM than in BCM. Furthermore, the experimental group performing the 5-day HAM training showed significant improvements in the performance of Stroop, Gabor, Reading, and Math tests after the training. Although there was uniform enhancement in the performance of these tests observed among the HAM group, the control group just had significant improvements in the Math and Stroop tests and even a reduction in the SART performance after the 5-day BCM training. The experimental group showed significantly greater improvement in the SART after 5 days of HAM than did the controlled BCM group. The HAM group tended to show greater improvements in the Stroop, Gabor, Math, and Reading tests than did the BCM group, although these improvements were not significant statistically. These outcomes after only 5 days of training suggested the potential of HAM for more considerable improvement in attention performance as the training sessions increased.

The desired meditative state stresses a balanced state of relaxation while focusing on attention, and this state is considered an essential factor in improving the efficacy of exercise on cognitive function (Walsh and Shapiro, 2006; Macdonald et al., 2013). The HAM paradigm was designed to help novices maintain this balanced state in terms of the following aspects. First, the paradigm integrated several vital components of meditation, including deep breathing, mental imagery, body sensations, and mindfulness training. These components have shown broad positive effects on attention control and emotion regulation in previous studies (Tang et al., 2015). When performing HAM, practitioners breathed deeply, slowly, and stably and needed to focus attention on their fingers to keep synchronized with respiration. Such a process was expected to induce a state of restful alertness. Besides, the design of the two-hand response was also introduced to allow a comfortable and relaxed posture because most participants had reported unnatural posture for the one-hand response in the preliminary experiment.

Second, single focus is considered a critical factor that facilitates the efficacy of attention training (Lippelt et al., 2014). However, this factor might be at risk in the proposed paradigm, as HAM was a dual task of breath awareness and force control, and participants might need to switch attention between the two sub-tasks. Nevertheless, participants in this study scarcely reported that their attention on the breathing was distracted by the force control. Instead, they tended to report the breath focusing and the force control as one integrated task. For example, they reported in their post-questionnaires that “These two tasks seem to be one, and it is very natural,” “I imaged an energy flow circulating between my abdomen and fingertips,” and “I felt my body as a whole when performing the task.” Therefore, the dual-task design of HAM did not prevent practitioners from maintaining the desired state of relaxation and focusing. The improvement in the performance of attentional tests after the HAM training also supported that the design did not risk the effectiveness of HAM on attention enhancement.

Finally, we designed a relatively complicated paradigm for HAM. Precisely, participants needed to perform four progressive blocks (i.e., breath counting, positive mapping, negative mapping, and hybrid mapping) in each session. The breath-counting block first provided participants a brief relaxed period to help them get into the desired state gradually. The following three blocks were similar but had differences in the mapping mode, which was designed to improve the participant’s engagement in the practice. Besides, the paradigm of block 4 required to count and retain the number of respiratory cycles and thus combined meditation practice with working memory training. Given that working memory training also benefits attentional functions (Klingberg, 2010; Spencer Smith and Klingberg, 2015), this combination was expected to amplify the training effect over the use of only one of these components for novice meditators.

Many styles of meditation practice rely on thought control, such as focusing on the breath and paying attention to the present moment. However, the approach of controlling thoughts might be not optimal for novices because they have to struggle to control their thoughts from mind-wandering in the early stage of meditation. Compared with the traditional meditation practice stressing thought control, HAM required concentration on a specific response from the body, while reducing reliance on the control of thoughts, which may be more accessible for novices. Besides, compared with BCM, HAM required a higher cognitive load and thus inhibited participants from spontaneous and frequent mind-wandering. Considering that a high cognitive demand might be a potential factor that influences the efficacy of exercise on cognitive function (Ji et al., 2017, 2019), the higher cognitive load in HAM suggested a stronger training dose than pure breath awareness meditation and thus may accelerate the practitioner’s access to meditative states.

Because the HAM paradigm is suitable for novices and raises the possibility of amplifying the training effect, we hypothesized that a short period of training might benefit the self-regulation of attention networks. Although 5 days of training was indeed short for novices, we have reasons to expect that the 5-day HAM practice could lead to improvements in attention functions. Previous findings indicated that the 5-day meditation practice with the integrative body–mind training method induced significantly better attention and control of stress than in a control group given relaxation training (Tang et al., 2007). Our previous work also demonstrated that 5 days of training with a force-control task improved the efficiency of the executive attention networks (Wang et al., 2014). Raz et al. (2005) found changed brain processes even after a single meditation session. Besides, there existed viewpoints that the amount of time participants spent meditating each day, rather than the total number of hours of meditative practice over their lifetime, affects performance on attentional tasks (Chan and Woollacott, 2007). Taken together, a short (5 days) but intensive (1 h/day) training was conducted in this study. Nevertheless, we should note that for a short period of training, the high-quality practice was necessary for every session, and the experimental process needed to guarantee that each practice session achieved a good result. A longer period of practice is still needed in future research to investigate the long-term effect of HAM on attention functions.

Our previous study has validated that the synchronization between the force and respiration signals can be used as an objective marker of attentional state (Zheng et al., 2019). Thus, another advantage of the HAM paradigm might lie in the possibility of real-time monitoring of the attentional state. The monitoring allows novices to obtain real-time feedback of their brain state and, therefore, could promote the training by proper intervention or guidance. In the present study, the algorithm for computing Δ_TS_ had an average error of about 50 ms and indicated an overall accuracy of 87.5% because participants performed an approximately 400-ms Δ_TS_ during the training. The accuracy met the requirements for assessing the changes in attentional performance over time in this study. It should be noted that the current algorithm was executed offline, and how to improve the accuracy and speed of the algorithm for online calculation is needed to be investigated in further research.

Functional near-infrared spectroscopy findings in this study suggested that an increased brain activation could occur after the practice, which was consistent with most previous literature. We found significantly increased oxy-Hb and decreased deoxy-Hb over the right PFC when performing ANT and SART in the posttests. There was also an increasing trend of oxy-Hb in the right PFC during the resting state. These findings support the assumption that meditation beginners who need to overcome habitual ways of internally reacting to one’s unrelated thoughts might show a greater prefrontal activation (Allen et al., 2012). Achieving the focused meditative state involves the attentional control effort in the early stage of meditation, thus activating the prefrontal regions. It should be noted that this study used the task-level (block-level) design for the fNIRS data analysis. Future research is advised to investigate event-related responses within trials of the testing task, as well as during the meditation practice, which might provide more information regarding brain attention functions. Neural recordings for the control group are also necessary to understand the differences between HAM and traditional meditation types.

Moreover, although the FC analyses were limited in terms of the small sample size, this study suggested consistent findings with previous studies showing that meditation increased certain synchronous activities within default mode network (DMN) (Jang et al., 2011). Following the training, we observed a stronger FC between the medial PFC and SMC at resting state. Given that the medial PFC and inferior parietal lobule are considered key regions of the DMN involved in self-referential processing (Northoff et al., 2006; Sajonz et al., 2010), the stronger connectivity may be interpreted as increased self-monitoring and cognitive control over the DMN function (Brewer et al., 2011). Besides, we observed a reduced FC between most channels over the PFC and SMC in the post-SART, as well as between some channels of the PFC and temporal cortex in the post-ANT. Similar findings were shown in previous fMRI studies. For example, one cross-sectional study on pain processing demonstrated decreased connectivity of executive and pain-related brain regions in meditators (Grant et al., 2011). We accordingly speculated that the reduced coupling at the tasking state might be associated with increased attentional control because participants did not have to pay so much effort for the stimulus monitoring and response inhibition after the training. Nevertheless, whether reduced coupling between these brain regions indicates improved attentional regulation needs further investigation with a larger number of subjects. Besides, the current fNIRS system did not provide the accurate neuro-navigation information corresponding to the brain anatomical structure; thus, findings regarding more precision regions of interest were not discussed in this study. Further FC analysis for more precision brain regions such as the dorsolateral PFC might provide additional information.

On the basis of these behavioral and fNIRS results, we surmised that HAM affected brain attentional functions positively by activating the attention network, SMC, and the regions related to working memory. The effective activations were obtained by the specific design of the HAM paradigm that involved body sensations, muscle control, attention switching within the internal body, and working memory practice. HAM incorporated respiratory perception with force control, requiring somatosensory attention as a physiological foundation and thus provided a useful way for novices to obtain a series of key meditation skills, including (i) how to feel directly when mind has wandered from its sensory focus, (ii) how to control somatic attention to compete with internally focused rumination, (iii) how to obtain familiarity with the fluctuations of sensations of breathing and forces, and (iv) how to control the attentional spotlight to shift from one to another flexibly. Together, these enhanced skills might become a crucial early stage of attention enhancement (Kerr et al., 2013).

## Conclusion

This study adds to the literature demonstrating the potential value of human haptic channels in cognitive training. Preliminary results demonstrate the improvements in behavioral performance and brain activation following 5 days of practice, which provides evidence for the effectiveness of HAM on attention enhancement in the early stage of meditation learning. On the basis of the present work, future longitudinal studies with larger subject populations and controlled neural recordings will be necessary to validate the conclusions further. Another possible place for future research is assessing the moment-to-moment attentional state during the practice by online computing of the synchronization between breathing and force signals. The online method can be used to link fNIRS measurements to attentional performance and possibly develop a real-time neurofeedback paradigm.

## Data Availability Statement

The raw data supporting the conclusions of this manuscript will be made available by the authors, without undue reservation, to any qualified researcher.

## Ethics Statement

Experiments were approved by the State Key Laboratory of Virtual Reality Technology and Systems of China. Written informed consents were obtained from all participants.

## Author Contributions

Y-LZ and D-XW designed the research plan, organized the study, and wrote the manuscript. Y-RZ and D-XW administered the experiments. All authors coordinated the data analysis, interpreted the data, discussed the results, and edited the manuscript.

## Conflict of Interest

The authors declare that the research was conducted in the absence of any commercial or financial relationships that could be construed as a potential conflict of interest.

## Footnotes

1 http://homer-fnirs.org/
